# Supercritical CO_2_ extraction of onion seed oil and its synergistic effect with gentamicin against bacterial strains

**DOI:** 10.1039/d6ra04660f

**Published:** 2026-10-20

**Authors:** Selma Mestour, Abdeslam-Hassen Meniai, Sarra Melloul, Arif Mermer, Saban Tekin

**Affiliations:** a Process Engineering Laboratory, University of Constantine 3 Algeria meniai@yahoo.fr; b Experimental Medicine Application and Research Center, University of Health Sciences Istanbul Türkiye arif.mermer@sbu.edu.tr; c Faculty of Pharmacy, University of Health Sciences Istanbul Türkiye; d University of Health Sciences, Department of Biotechnology Istanbul Türkiye

## Abstract

Antibiotic resistance represents a major global health challenge, requiring innovative strategies to enhance the effectiveness of existing antimicrobial agents. In this study, onion seed oil was extracted using supercritical CO_2_ and evaluated for its potential to improve the antibacterial activity of the antibiotic gentamicin. The oil was characterized through physicochemical analyses, including FTIR spectroscopy, thermogravimetric analysis (TGA), and scanning electron microscopy (SEM). Antibacterial activity was tested against *Staphylococcus aureus* and *Escherichia coli* using the disk diffusion method. The results demonstrated that onion seed oil exhibited significant antibacterial activity and enhanced the efficacy of gentamicin when used in combination. The synergistic interaction increased inhibition zone diameters by up to 20.4% for *S. aureus* and 22.95% for *E. coli*, even at low extract concentrations. These findings suggest that supercritical CO_2_-extracted onion seed oil may serve as a promising natural adjuvant to improve antibiotic performance and combat bacterial resistance.

## Abstract 2

Supercritical CO_2_-extracted onion seed oil enhanced gentamicin activity against *S. aureus* and *E. coli*, increasing inhibition zones by up to 20% and 23%, respectively, and supporting its use as a natural antibiotic adjuvant.

## Graphical abstract

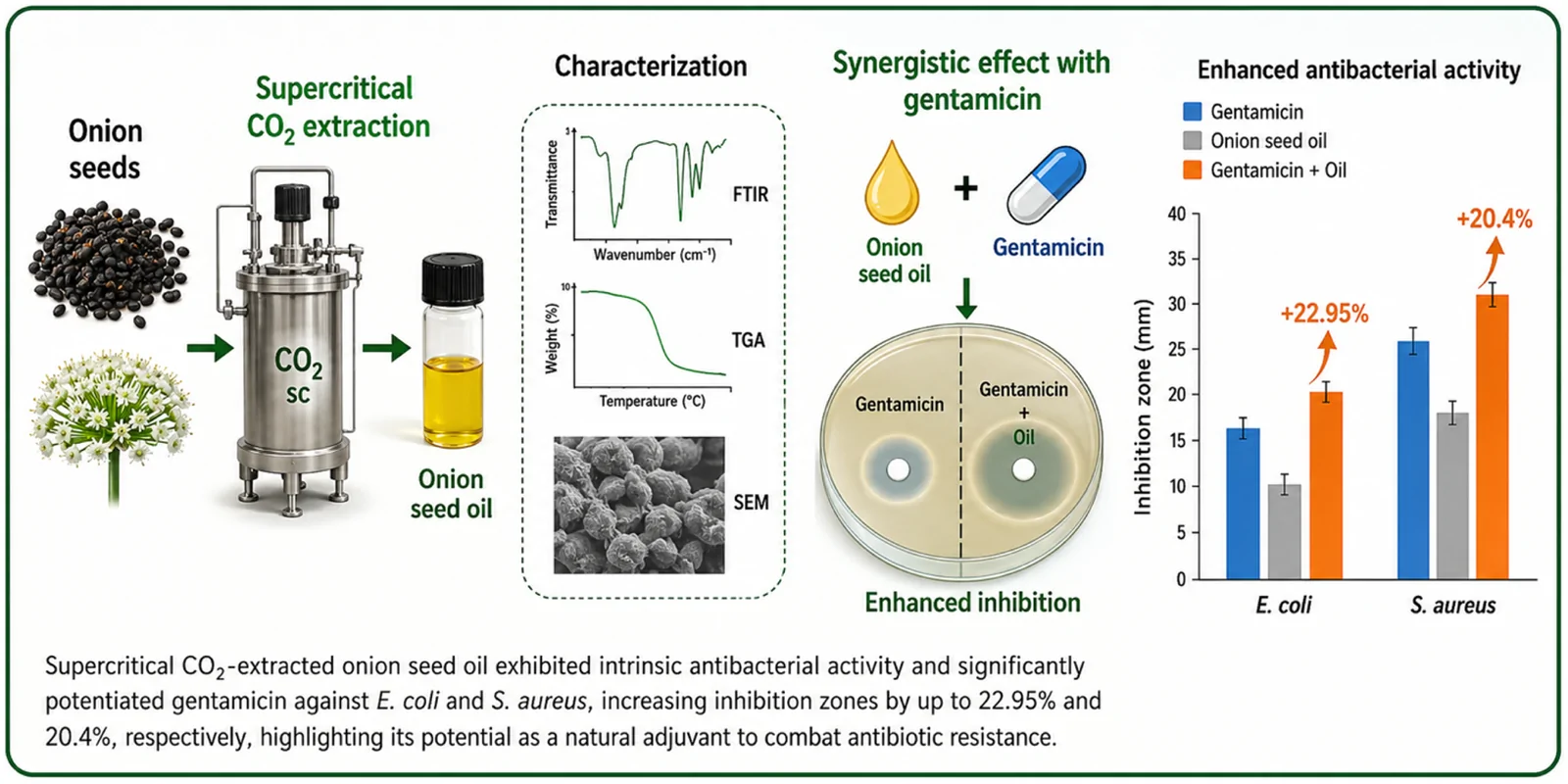

## 1. Introduction

Antimicrobial resistance has become a major global health challenge, significantly reducing the effectiveness of existing antibiotic therapies. Over the past decades, the number of bacterial pathogens resistant to multiple antimicrobial agents has increased dramatically, making the treatment of infectious diseases more difficult and costly.^1–3^ This growing resistance is driven by several mechanisms, including genetic mutations, enzymatic inactivation of drugs, modification of drug targets, and horizontal gene transfer between microorganisms.^4^ The misuse and overuse of antibiotics in human medicine, veterinary practice, and agriculture have further accelerated this phenomenon. It has even been estimated that antimicrobial resistance could cause up to 10 million deaths annually by 2050 if effective solutions are not developed.^5^

To address this challenge, several strategies have been proposed, including the development of new antimicrobial agents, improved infection control, and the use of combination therapies. In particular, combining conventional antibiotics with plant-derived bioactive compounds has emerged as a promising approach to enhance antibacterial efficacy and overcome resistance mechanisms.^6,7^ Medicinal plants are rich in secondary metabolites such as terpenes, flavonoids, polyphenols, and alkaloids, many of which exhibit antimicrobial and resistance-modifying properties.^7^ Previous studies have shown that natural compounds can improve antibiotic activity by increasing bacterial membrane permeability, inhibiting efflux pumps, or interfering with microbial metabolic pathways.^8,9^ For example, synergistic interactions between essential oils and antibiotics have been reported against several pathogenic bacteria, including *Staphylococcus aureus*.^10,11^

In parallel, the extraction technique used to obtain plant bioactive compounds plays a crucial role in determining their composition and biological activity. Among the available techniques, supercritical CO_2_ extraction has attracted considerable interest as a green and efficient method for recovering valuable natural products. In contrast to conventional solvent-based extraction, supercritical CO_2_ extraction can avoid the use of conventional organic solvents such as hexane, thereby reducing concerns related to solvent residues, flammability, solvent handling, and downstream solvent removal. In addition, CO_2_ can be readily separated from the extract by depressurization, minimizing the risk of residual organic solvents in the final extract.^12–14^ However, supercritical CO_2_ extraction requires elevated pressures and should therefore not be considered a low-pressure process. Nevertheless, it can generally be operated at relatively low or moderate temperatures, which may help limit the thermal degradation or oxidation of temperature-sensitive constituents. Furthermore, the density and solvent properties of supercritical CO_2_ can be adjusted by varying pressure and temperature, providing a degree of control over extraction performance and selectivity. Other operating parameters, including CO_2_ flow rate, particle size, extraction time, and the use of co-solvents, can also influence extraction efficiency and the composition of the resulting extracts.^14^ Supercritical CO_2_ extraction is particularly attractive for seed oils and other lipid-rich plant matrices because the relatively non-polar character of CO_2_ favors the extraction of non-polar and lipophilic constituents, while its tunable solvent properties allow the extraction process to be adjusted according to the target compounds.^15^ Nevertheless, its limited affinity for highly polar phytochemicals means that polar co-solvents such as ethanol may be required when the recovery of polar compounds is targeted.^13,14^

The application of supercritical CO_2_ to seed-oil extraction has been reported for various plant sources. Chen *et al.* obtained an optimized extraction yield of 24.36% for apple seed oil at 41 MPa and 56 °C.^16^ For *Nigella sativa* seed oil, extraction yields ranging from 6.13% to 31.15% were reported depending on the operating conditions, with pressure identified as the most influential parameter.^17^ Similarly, supercritical CO_2_ extraction of safflower seed oil resulted in a predicted optimum yield of 26.47% at 450 bar and 68.49 °C.^18^ These studies demonstrate the applicability of supercritical CO_2_ for recovering oils from different seed matrices and highlight the importance of extraction conditions in determining the yield and characteristics of the obtained oils.

Onion seeds (*Allium cepa* L.) constitute an interesting plant matrix because of their lipid content and bioactive constituents, as well as their reported nutritional and biological properties.^19,20^ Previous studies on onion seeds have mainly focused on their chemical composition, nutritional characteristics, and biological properties. In this context, our previous study demonstrated the feasibility of extracting the oil fraction from onion seeds using supercritical CO_2_ and optimized the process using a Box–Behnken design. The optimum conditions were 250 bar, 57 °C, a particle size of 0.25 mm, and a CO_2_ flow rate of 50 g min^−1^, resulting in an extraction yield of 24.36%.^21^

Beyond extraction performance, the interest in this oil also lies in the potential valorization of its constituents for biological applications. Although the antioxidant and antimicrobial properties of onion seeds and their constituents have been reported in the literature,^19,20^ information specifically addressing the antibacterial activity of onion seed oil obtained by supercritical CO_2_ extraction and its use in combination with a conventional antibiotic remains limited. This question is particularly relevant in the context of antimicrobial resistance, where the combination of natural products with conventional antibiotics is being investigated as a strategy to enhance antibacterial efficacy. Since the composition of plant extracts is closely influenced by extraction conditions, the biological evaluation of an oil obtained under optimized extraction conditions can provide complementary information to its chemical characterization and extraction performance.

The novelty of the present study therefore lies in evaluating the antibacterial activity of onion seed oil obtained by supercritical CO_2_ extraction and investigating its interaction with gentamicin against *Staphylococcus aureus* and *Escherichia coli*. The study further investigates the biological properties of the extracts by determining their total phenolic, flavonoid, and hydrolysable tannin contents, together with their antioxidant activity. This approach complements our previous work on extraction optimization and oil characterization by exploring the biological potential of the obtained oil and its potential value as a natural adjuvant to conventional antibiotic therapy.

## 2. Materials and methods

### 2.1 Plant material

Onion seeds (*Allium cepa* L.) were collected during the harvest season from the Oued Souf region in southeastern Algeria, an area known for its large-scale onion production. The seeds were cleaned and stored under dry conditions prior to extraction.

### 2.2 Chemicals and reagents

All reagents used in this study were of analytical grade. Folin–Ciocalteu reagent, gallic acid, aluminum trichloride (AlCl_3_), sodium nitrite (NaNO_2_), quercetin, sodium carbonate (Na_2_CO_3_), potassium iodate, tannic acid, methanol, dimethyl sulfoxide (DMSO), and Trolox were obtained from Sigma-Aldrich or VWR Chemicals. Mueller–Hinton agar and Sabouraud agar were used for microbiological analyses.

### 2.3 Supercritical CO_2_ extraction of onion seed oil

Onion seed oil was extracted using a laboratory-scale supercritical CO_2_ extraction system (Separex-4219, Separex, Champigneulles, France) installed at the Environmental Process Engineering Laboratory, University of Constantine 3, Algeria. Carbon dioxide (CO_2_, 99.99% purity), supplied by Air Liquide, Algeria, was used as the extraction solvent. The main technical specifications of the extraction vessel and separators are summarized in Table 1.

**Table 1:** Main technical specifications of the supercritical CO_2_ extraction apparatus

Parameter	Extraction vessel	Separators
Number	1	2, connected in series
Internal volume	2 L	0.5 L each
Empty weight	47 kg	9 kg each
Nominal pressure	800 bar	200 bar
Maximum temperature	200 °C	150 °C
Test pressure	1144 bar	325 bar

A schematic representation and a photograph of the extraction system are presented in Fig. 1.

**Fig. 1:**
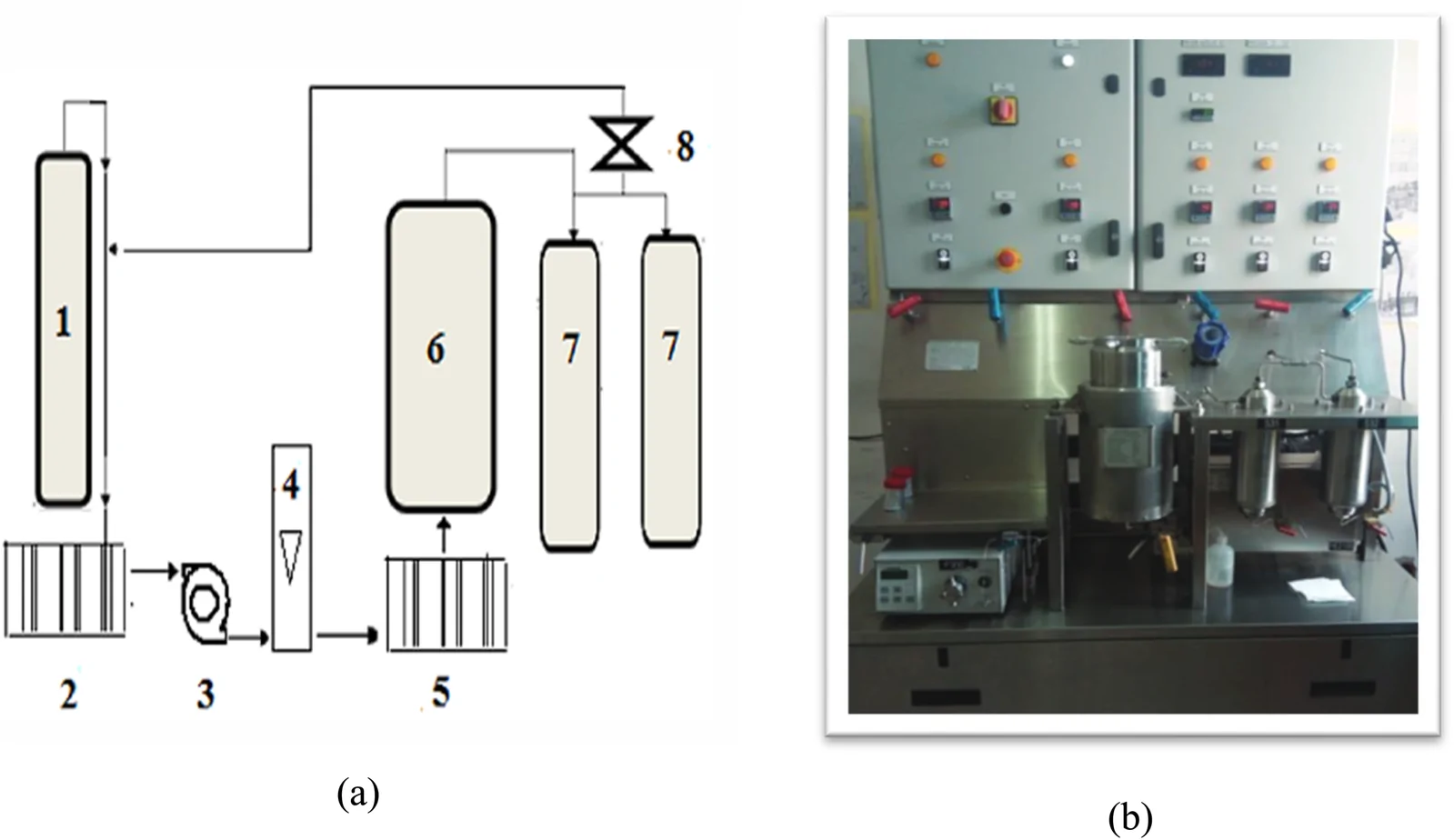
Supercritical CO_2_ extraction system used for onion seed oil extraction: (a) schematic representation of the system and its main components; (1) CO_2_ storage tank; (2) cooler; (3) high-pressure pump; (4) flowmeter; (5) heater unit; (6) extractor; (7) separators; (8) expansion valve; (b) photograph of the Separex-4219 apparatus.

Prior to extraction, the onion seeds were cleaned to remove foreign materials, ground, and sieved to obtain three particle-size fractions (0.25, 0.55, and 0.85 mm). For each experimental run, 40 g of ground onion seeds were introduced into the extraction vessel.

With reference to Fig. 1, liquid CO_2_ from the storage tank (1) was first cooled in the cooler (2) and then pressurized using the high-pressure pump (3) to a pressure above the critical pressure of CO_2_ (7.38 MPa). The pressurized CO_2_ was subsequently heated to the selected extraction temperature before entering the extraction vessel (6), which contained 40 g of the prepared ground onion seeds. The heating unit (5) was used to maintain the required extraction temperature. Under these conditions, supercritical CO_2_ passed through the plant matrix and solubilized the extractable oil fraction. The resulting CO_2_–oil mixture leaving the extraction vessel (6) was then directed to the two separators connected in series (7), where pressure reduction decreased the solubility of the extracted oil in CO_2_, allowing the oil fraction to be separated and collected. Finally, the CO_2_ stream was expanded through the expansion valve (8), recovered, and recycled for subsequent extraction runs.

The extraction process was conducted according to a four-factor Box–Behnken response surface design, selected to evaluate the linear, quadratic, and interaction effects of temperature (*T*), pressure (*P*), particle size (*D*), and CO_2_ flow rate (*Q*) within the investigated experimental domain, following the experimental design previously reported by Mestour *et al.*^21^ The experimental ranges were 35–65 °C for temperature, 150–250 bar for pressure, 0.25–0.85 mm for particle size, and 50–100 g min^−1^ for CO_2_ flow rate. Each extraction was performed for 3 h under the corresponding experimental conditions. A total of 27 experimental runs were performed, including three replicates of the central point (50 °C, 200 bar, 0.55 mm, and 75 g min^−1^). The extraction temperature, pressure, and CO_2_ flow rate were controlled throughout each run using the instrumentation of the extraction system.

Following extraction, the collected oil extracts were recovered from the separators and stored at low temperature until further analysis.

### 2.4 Determination of bioactive compounds

#### 2.4.1 Total phenolic content

The total phenolic content (TPC) of the onion seed oil extracts was determined using the Folin–Ciocalteu method according to Singleton and Rossi.^22^ Briefly, 20 µL of extract was mixed with 100 µL of diluted Folin–Ciocalteu reagent (1 : 10) and 75 µL of sodium carbonate solution (7.5%). The mixture was incubated in the dark for 2 h at room temperature. Absorbance was measured at 765 nm using gallic acid as the standard. Results were expressed as mg gallic acid equivalents per gram of extract (mg GAE per g E).

#### 2.4.2 Total flavonoid content

Total flavonoid content (TFC) was determined using the aluminum chloride colorimetric method. A volume of 50 µL of extract was mixed with 20 µL of NaNO_2_ (5%), followed by incubation for 5 min. Then 20 µL of AlCl_3_ (10%) was added and incubated for 6 min. Finally, 100 µL of NaOH (4%) was added and the absorbance was measured at 430 nm. Quercetin was used as the calibration standard, and results were expressed as mg quercetin equivalents per gram of extract (mg QE per g E).

#### 2.4.3 Hydrolysable tannins

Hydrolysable tannins were determined using the potassium iodate assay. Briefly, 50 µL of extract (1 mg mL^−1^) was mixed with 150 µL of potassium iodate solution (2.5%). After 15 min of incubation, the absorbance was measured at 550 nm. Tannic acid was used as a reference compound and the results were expressed as mg tannic acid equivalents per 100 g dry weight (TAE mg per 100 g DW).

### 2.5 Antioxidant activity

The antioxidant activity of the onion seed oil extracts was evaluated using two assays: DPPH radical scavenging activity and ABTS radical scavenging activity. These methods are based on the ability of antioxidant compounds to donate electrons or hydrogen atoms to neutralize free radicals.^23^

### 2.6 Antibacterial activity

The antibacterial activity of onion seed oil extracts was evaluated against *Escherichia coli* ATCC 25922 and *Staphylococcus aureus* ATCC 25923 using the disk diffusion method. Mueller–Hinton agar plates were inoculated with bacterial suspensions and sterile filter paper discs (6 mm diameter) impregnated with 20 µL of oil extract were placed on the agar surface.

A negative control consisting of sterile filter paper discs impregnated with 20 µL of methanol, used as the diluting solvent for the oil extracts, was included under the same experimental conditions to verify that the solvent itself did not exhibit antibacterial activity. The plates were incubated at 37 °C for 24 h, and the antibacterial activity was assessed by measuring the diameter of the inhibition zones (mm) around the discs.

### 2.7 Determination of minimum inhibitory concentration (MIC)

The minimum inhibitory concentration (MIC) of the onion seed oil extracts was determined using serial dilutions of the extracts. Different concentrations were prepared and tested against *S. aureus* and *E. coli* using the disk diffusion method. The MIC was defined as the lowest concentration capable of inhibiting visible bacterial growth.

### 2.8 Combination of gentamicin and onion seed oil

To investigate the potential synergistic effect, mixtures of gentamicin at different concentrations and onion seed oil at its minimum inhibitory concentration were prepared. Equal volumes of the antibiotic solution and the oil extract were combined and applied to sterile filter paper discs. The antibacterial activity of the mixtures was then evaluated against the bacterial strains using the disk diffusion method.

### 2.9 FTIR analysis

Fourier transform infrared (FTIR) spectroscopy was used to characterize the chemical composition of onion seed oil extracts and to evaluate their compatibility with gentamicin. The spectra were analyzed to identify characteristic functional groups and to detect possible physicochemical interactions between the oil and the antibiotic. FTIR spectra were recorded using a Thermo Scientific Nicolet iS50 FTIR spectrometer attenuated total reflectance (ATR) accessory. The spectra were collected over the wavenumber range of 4000–400 cm^−1^ at a spectral resolution of 4 cm^−1^ by averaging 16 scans for each sample.

### 2.10 Thermogravimetric analysis (TGA)

Thermogravimetric analysis was performed to investigate the thermal behavior of onion seeds before and after supercritical CO_2_ extraction. Samples were heated progressively while recording mass loss as a function of temperature. This analysis allowed the evaluation of extraction efficiency and the identification of the main thermal degradation stages of the seed components. Thermogravimetric analysis (TGA) was performed using a PerkinElmer TGA 8000 thermogravimetric analyzer. Approximately 3 mg of each sample was placed in an open alumina pan and heated from 50 to 800 °C at a heating rate of 10 °C min^−1^ under a continuous nitrogen atmosphere. The purge gas flow rate was maintained at 40 mL min^−1^ throughout the analysis.

### 2.11 Scanning electron microscopy (SEM)

Scanning electron microscopy was used to examine the microstructural changes in onion seeds before and after supercritical CO_2_ extraction. The morphological characteristics of the samples were analyzed to assess the structural modifications induced by the extraction process.

## 3. Results and discussion

### 3.1 Determination of total bioactive components

The contents of total phenolic compounds (TPC), total flavonoids (TFC), and hydrolysable tannins in onion seed oil extracts obtained under different supercritical CO_2_ extraction conditions are presented in Table 2.

**Table 2:** Extraction yield and bioactive composition of onion seed oil obtained under different operating conditions

Operating parameters					TPC (mg GAE per g E)	TFC (mg QE per g E)	HT (TAE mg per 100 g DW)	*Y*_exp_ (%)
Extract	*T* (°C)	*P* (bar)	*D* (mm)	*Q* (g min^−1^)				
1	35	150	0.55	75	—	84.588	134.882	17.876
2	65	150	0.55	75	12.923	89.98	212.529	11.604
3	35	250	0.55	75	—	69.686	20.471	18.047
4	65	250	0.55	75	13.02	89.49	51.451	18.253
5	50	200	0.25	50	—	66.157	31.353	24.320
6	50	200	0.85	50	15.666	75.667	25.863	21.199
7	50	200	0.25	100	7.627	75.961	7.627	22.241
8	50	200	0.85	100	69.686	43.804	69.686	18.09
9	35	200	0.55	50	—	118.706	234.098	22.017
10	65	200	0.55	50	31.157	142.529	1.843	20.664
11	35	200	0.55	100	—	60.961	22.627	23.161
12	65	200	0.55	100	4.686	97.824	32.039	18.473
13	50	150	0.25	75	—	100.765	151.255	19.854
14	50	250	0.25	75	36.157	132.039	94.98	24.350
15	50	150	0.85	75	9.392	99.392	21.647	16.411
16	50	250	0.85	75	—	69.98	42.039	22.625
17	35	200	0.25	75	—	108.412	129	24.039
18	65	200	0.25	75	6.843	136.451	82.529	21.974
19	35	200	0.85	75	7.431	173.608	67.529	19.371
20	65	200	0.85	75	23.216	136.255	80.765	17.408
21	50	150	0.55	50	15.863	138.902	78.706	21.407
22	50	250	0.55	50	8.51	109.294	84.980	22.695
23	50	150	0.55	100	10.275	117.725	42.431	19.902
24	50	250	0.55	100	6.353	124.196	144.196	22.210
25	50	200	0.55	75	28.25	81.353	40.65	20.968
26	50	200	0.55	75	27.5	80.63	40.863	18.174
27	50	200	0.55	75	27.039	79.686	66.941	20.455
Opt.	57	250	0.25	50	**2.529**	**88.314**	**58.314**	**24.361**

The results show that the concentration of bioactive compounds varies considerably depending on the extraction parameters, particularly temperature, pressure, particle size, and CO_2_ flow rate. The total phenolic content ranged from 2.53 to 69.69 mg GAE per g extract, while the flavonoid content varied between 43.80 and 173.61 mg QE per g extract. These variations indicate that the extraction conditions significantly influence the recovery of phenolic compounds from the onion seed matrix.

Temperature appeared to have a positive influence on the extraction of phenolic and flavonoid compounds. For instance, increasing the temperature from 35 to 65 °C resulted in an increase in both TPC and TFC values under comparable operating conditions. The increase observed at higher temperatures may result from the combined effects of CO_2_ density, solute vapor pressure, and mass transfer. The balance between these effects determines the solubility and extraction efficiency of bioactive compounds under the investigated conditions.

In contrast, the influence of pressure and CO_2_ flow rate on the phenolic and flavonoid contents was less consistent. Increasing pressure generally increases the density of supercritical CO_2_ and consequently its solvent strength, thereby enhancing the solubility of extractable constituents.^24^ However, pressure can also modify extraction selectivity because compounds with different polarity, molecular size, and affinity for supercritical CO_2_ do not respond equally to changes in solvent density. Supercritical CO_2_ is relatively non-polar and therefore generally exhibits greater affinity for non-polar and lipophilic constituents than for more polar phenolic compounds.^24,25^ In lipid-rich matrices such as onion seeds, increasing pressure may therefore promote the co-extraction of non-polar and lipophilic constituents, thereby modifying the relative composition of the recovered extract. Consequently, the relative proportion of phenolics in the extract may decrease even when the overall extraction yield increases. This interpretation is particularly relevant in the present study because TPC and TFC are expressed per gram of extract; therefore, increased co-extraction of lipids or other non-phenolic constituents could result in lower apparent TPC or TFC values through a dilution effect. In addition, increasing fluid density at elevated pressure may be accompanied by changes in diffusivity and mass-transfer behavior, which can further influence the recovery of individual compounds.^24^ Therefore, the influence of pressure on phenolic and flavonoid contents likely reflects a balance between increased solvent strength, compound-specific solubility, co-extraction of other constituents, and mass-transfer phenomena, which may explain the non-monotonic variations observed under the investigated conditions.

Hydrolysable tannins are a group of phenolic compounds found in a wide variety of plants, including fruits, nuts, seeds, bark, and other plant tissues. The hydrolysable tannin content of onion seed extracts is presented in Table 2. The hydrolysable tannin content ranged from 1.843 to 234.098 mg TAE per 100 g dry weight (DW). These results suggest that the extraction of bioactive compounds is strongly influenced by the operating conditions. Although the hydrolysable tannin content varied considerably with the extraction conditions, no simple monotonic relationship with a single operating parameter (temperature, pressure, particle size, or CO_2_ flow rate) was observed. Instead, the results indicate that tannin recovery is governed by the combined effects of these operating parameters, which influence the solubility and mass transfer of these relatively polar compounds during supercritical CO_2_ extraction. Onion seed extracts proved to be a particularly valuable source of hydrolysable tannins compared with other plant materials, with a maximum value exceeding the 120.8 mg TAE per 100 g DW reported for the leaf tissues of *Terminalia ferdinandiana*,^26^ thereby confirming the high tannin content obtained in the present study. Hydrolysable tannins are an important class of plant tannins whose antibacterial properties have been widely reported. Several mechanisms have been proposed to explain this activity, including disruption of membrane integrity, interactions with bacterial proteins and enzymes, and interference with cellular processes such as bacterial adhesion and enzymatic activity. In addition, some hydrolysable tannins have been reported to act synergistically with conventional antibiotics.^27,28^ In the present study, the relatively high hydrolysable tannin contents observed in some onion seed extracts indicate a notable presence of this class of bioactive compounds. Considering the antibacterial properties reported in the literature, these compounds may contribute to the antibacterial activity observed. This contribution is likely part of an overall effect resulting from the combined action of hydrolysable tannins with other bioactive constituents present in the extracts, as well as from possible interactions among these different classes of compounds.

To better highlight the combined effects of temperature and pressure on bioactive compounds, contour plots were generated for total phenolic content (TPC), total flavonoid content (TFC), and hydrolysable tannins (HT), at a fixed particle size of 0.55 mm and a CO_2_ flow rate of 75 g min^−1^ (Fig. 2). The plots reveal distinct nonlinear responses depending on the parameter considered.

**Fig. 2:**
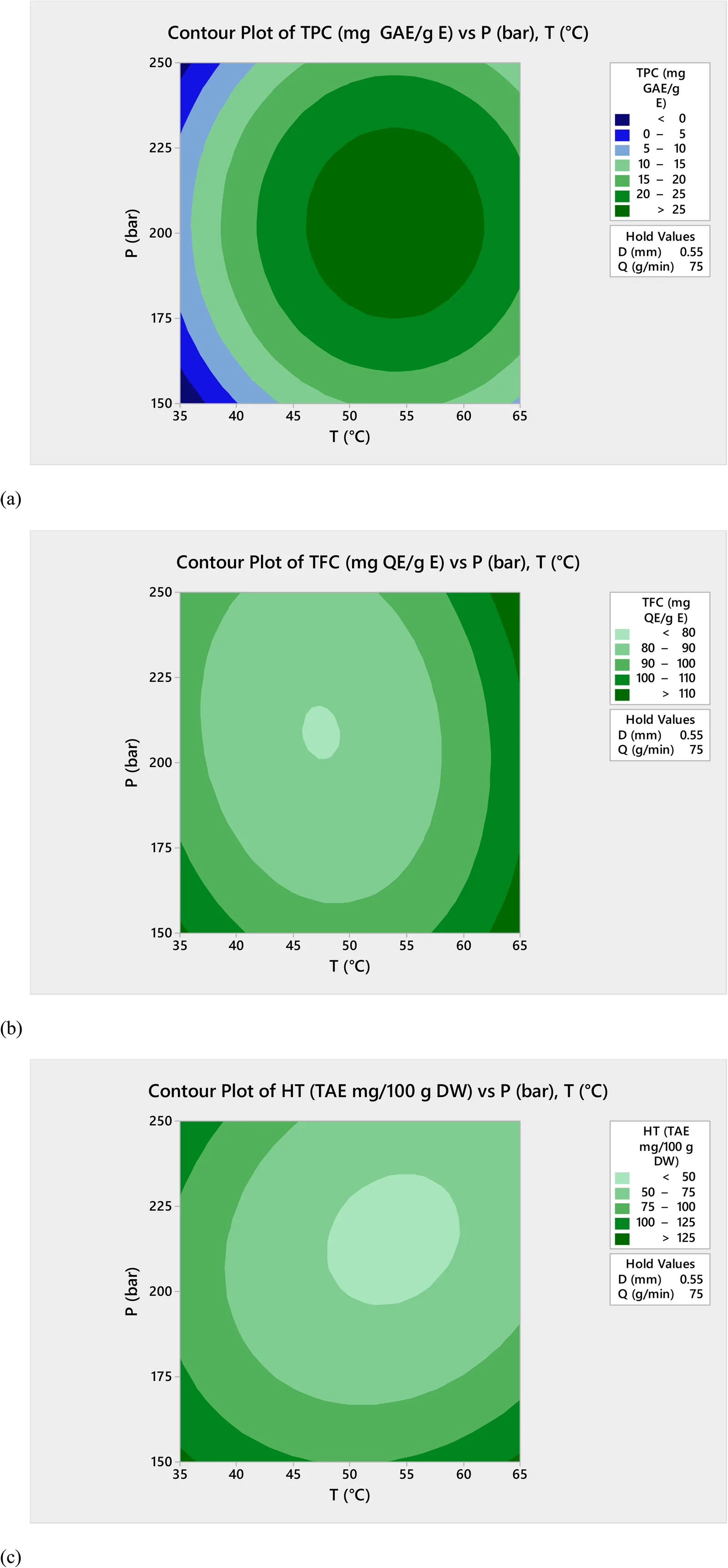
Contour plots showing the combined effects of temperature and pressure on (a) total phenolic content (TPC), (b) total flavonoid content (TFC) and (c) hydrolysable tannin content (HT).

For TFC, the lowest values are observed at intermediate temperatures, around 45–52 °C, whereas an increase occurs outside this range, particularly at higher temperatures, suggesting a stronger influence of temperature than pressure.

A similar trend is observed for HT, with the lowest contents occurring approximately between 50 and 58 °C and 200 and 230 bar. Higher values are observed outside this region, particularly at low pressures and under certain combinations of low temperature and high pressure.

In contrast, TPC exhibits a different pattern, increasing toward the central region of the experimental domain. The highest values are observed approximately between 50 and 60 °C and 180 and 225 bar, with a maximum region around 55 °C and 200 bar. This difference may be related to the distinct chemical characteristics and extraction affinities of the phenolic fractions assessed by the three assays. Although TPC, TFC, and HT are all related to phenolic constituents, they are determined using different colorimetric approaches and do not necessarily represent identical chemical fractions. The Folin–Ciocalteu assay provides an overall estimation of reducing phenolic constituents, whereas the TFC and HT assays preferentially assess flavonoid-related and hydrolysable tannin fractions, respectively. Differences in chemical structure, polarity, solubility, and affinity for supercritical CO_2_ may therefore result in different extraction behaviors under changing temperature and pressure conditions. The opposite trend observed for TPC compared with TFC and HT may consequently reflect the selective recovery of different phenolic fractions within the investigated experimental domain. Overall, the curvature and non-monotonic patterns observed in the contour plots demonstrate that the extraction responses are not governed by simple linear effects of temperature and pressure. Within the investigated experimental domain, these trends are consistent with the quadratic response-surface approximation used in the Box–Behnken design. However, the observed curvature should be interpreted as a local empirical representation of the extraction response rather than as a direct representation of the underlying thermodynamic behavior of supercritical CO_2_. The combined influence of temperature and pressure therefore depends on the type of bioactive compounds, with intermediate conditions favoring higher TPC but generally lower TFC and HT contents.

### 3.2 Total antioxidant activity

The antioxidant activity of onion seed oil extracts was evaluated using two different assays: DPPH radical scavenging activity and ABTS radical scavenging activity. These methods assess the capacity of antioxidant compounds to neutralize free radicals by donating electrons or hydrogen atoms.^23^

The results showed low antioxidant activity under all investigated extraction conditions, with IC_50_ values exceeding 4000 µg mL^−1^ in both assays. These results indicate a weak antioxidant capacity of the onion seed oil extracts, regardless of the extraction conditions applied. This finding is consistent with the results reported by Yalcin and Kavuncuoglu, who evaluated ethanol extracts obtained by cold extraction of onion seed and reported DPPH radical inhibition values ranging from 4.59 to 38.26% at a concentration of 100 000 µg g^−1^, indicating relatively low antioxidant activity.^29^

### 3.3 Antibacterial activity of onion seed oil

The antibacterial activity of the onion seed oil extracts obtained under different supercritical CO_2_ extraction conditions was evaluated against *Escherichia coli* and *Staphylococcus aureus*. The inhibition zone diameters obtained for the different extracts are presented in Table 3.

**Table 3:** Inhibitory zone diameter values (mm) obtained for the antibacterial activity of pure onion seeds oil

Extract	*E. coli*	*S. aureus*
	Medium diameter (mm)	Medium diameter (mm)
01	18.5	31
02	16.5	22.5
03	13	30.5
04	16.5	38.5
05	23	25
06	**37.5**	26.5
07	11	14
08	20.5	29
09	12.5	14.5
10	17.5	16.5
11	15.5	30
12	14	30.5
13	25.5	**39**
14	15	29
15	12	17.5
16	—	14.5
17	13	22
18	21.5	33
19	21.5	33.5
20	—	18.5
21	19	20.5
22	14	27.5
23	11.5	11.5
24	12	27
25	11.5	20.5
26	11.5	19.5
27	18	19
Optimum	27	36
Methanol (negative control)	0	0

The negative control (methanol), used as the diluting solvent for the extracts, showed no inhibition zone against either bacterial strain, confirming that the solvent itself did not contribute to the observed antibacterial activity. The results showed that onion seed oil exhibited antibacterial activity against both bacterial strains, with inhibition zone diameters ranging from 11 to 37.5 mm for *E. coli* and from 11.5 to 39 mm for *S. aureus*. The highest inhibition zones were obtained with extract 06 against *E. coli* and extract 13 against *S. aureus*. For comparison, Nadon *et al.* reported inhibition zones of 9.00 ± 0.63 mm against *E. coli* and 11.50 ± 0.84 mm against *S. aureus* for Makwaen essential oil obtained by supercritical fluid extraction (Nadon *et al.*, 2023 (ref. 31)). Similarly, supercritical CO_2_ extracts of sea buckthorn pomace produced inhibition zones ranging from 16.25 ± 0.35 to 16.75 ± 0.35 mm against *E. coli* and from 21.40 ± 0.28 to 27.35 ± 0.35 mm against *S. aureus*.^30^ These comparisons indicate that the inhibition zones observed for the onion seed oil extracts overlapped with, and in several cases exceeded, those reported for other plant extracts obtained by supercritical fluid extraction. In particular, the maximum inhibition zones observed for the onion seed oil extracts were substantially higher than those reported by Nadon *et al.* and for both bacterial strains.^31^ The considerable variation in antibacterial activity among the onion seed oil extracts may be related to differences in the composition of the extracts resulting from the different extraction conditions.

Previous studies have shown that supercritical CO_2_ extraction conditions can influence both the chemical composition and the biological activity of plant extracts. Mesomo *et al.* demonstrated that variations in extraction pressure and temperature affected the amounts of individual compounds recovered from ginger and resulted in differences in the antibacterial activity of the extracts.^32^ In particular, extracts obtained at higher pressure and temperature exhibited lower antibacterial activity than those obtained under other extraction conditions. Similarly, Arumugham *et al.* reported that temperature and pressure regulate the density and solvating properties of supercritical CO_2_, thereby influencing extraction yield, selectivity, and the biological properties of the extracted components.^33^ However, the effect of extraction temperature and pressure on antimicrobial activity cannot be generalized, as it may also depend on the nature of the plant matrix and the interactions between the extraction solvent and the plant material. Therefore, the differences observed among the onion seed oil extracts may, at least in part, reflect variations in the relative abundance of bioactive constituents resulting from the different extraction conditions. In addition, interactions among extract constituents may contribute to the overall antibacterial effect.

Differences in antibacterial response between the two strains may also be explained by structural variations between Gram-positive and Gram-negative bacteria. Gram-negative bacteria possess an outer membrane that can limit the penetration of hydrophobic compounds, whereas Gram-positive bacteria generally exhibit greater susceptibility to plant-derived oils.

The observed antibacterial activity may be related to the ability of lipophilic compounds present in onion seed oil to interact with bacterial cell membranes. Such interactions can lead to increased membrane permeability, disruption of cellular structures, and inhibition of essential metabolic processes, ultimately resulting in bacterial cell death. Similar mechanisms have been described for other plant essential oils.^34^

### 3.4 Minimum inhibitory concentration (MIC)

For the extracts that exhibited significant antibacterial activity against both *Escherichia coli* and *Staphylococcus aureus*, namely extract 1 (no. 1), extract 2 (no. 13), and extract 3 (optimum extract) from Table 3, tests were carried out at different concentrations to determine their minimum inhibitory concentration (MIC).

Fig. 3 shows that all three extracts followed the same trend, with the inhibition zone diameter increasing as the extract concentration increased against both bacterial strains. This trend was also observed at concentrations below 0.25 mg mL^−1^, confirming that onion seed oil retained inhibitory activity even at very low concentrations.

**Fig. 3:**
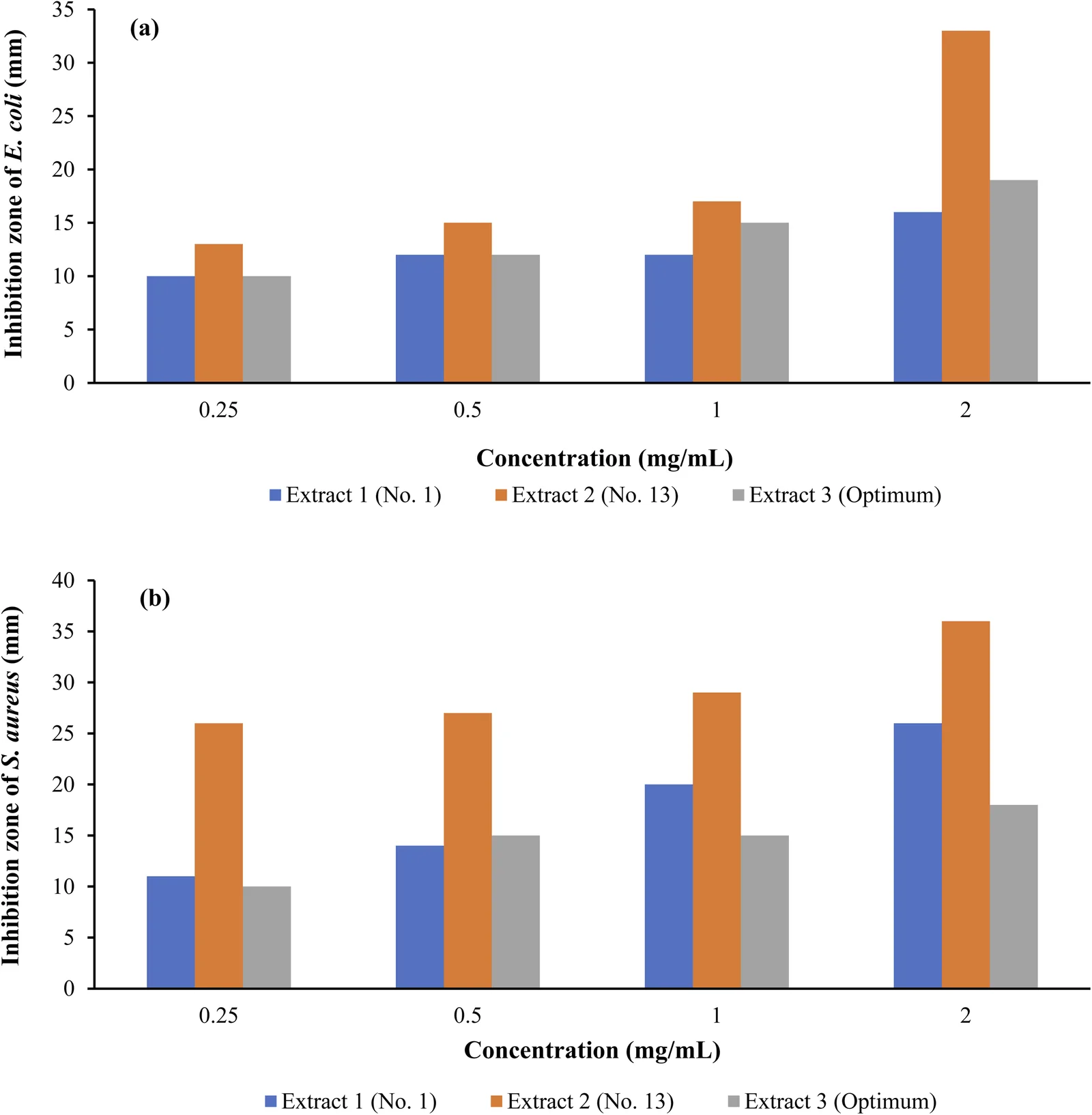
Inhibition zones of *E. coli* (a) and *S. aureus* (b) at different concentrations of onion seed oil extract.

Compared with other plant-derived oils reported in the literature, supercritical CO_2_-extracted onion seed oil exhibited superior antibacterial efficacy at a substantially lower concentration. At only 2 mg mL^−1^, the most active extract produced inhibition zones of 33 mm against *E. coli* and 36 mm against *S. aureus*.

In comparison, the essential oils of *Allium vineale* and *Allium sativum*, tested at 12.5 mg mL^−1^, showed considerably smaller inhibition zones of 6 and 7 mm against *E. coli* and 9 and 7 mm against *S. aureus*, respectively.^35^ Similarly, at a much higher concentration of 100 mg mL^−1^, baobab seed oil exhibited an inhibition zone of 19 mm against *E. coli*, while canary melon seed oil showed a 14 mm inhibition zone against *S. aureus*. Soxhlet-extracted onion seed oil, also tested at 100 mg mL^−1^, produced inhibition zones of 28 mm against *E. coli* and 21 mm against *S. aureus*.^36^

Thus, despite being tested at a concentration 6.25 to 50 times lower, the supercritical CO_2_-extracted onion seed oil produced larger inhibition zones than these previously reported oils, demonstrating its markedly higher antibacterial efficacy under the tested conditions.

### 3.5 Synergistic effect of gentamicin and onion seed oil

#### 3.5.1 Antibacterial activity of gentamicin

The antibacterial activity of gentamicin alone was evaluated against *Staphylococcus aureus* and *Escherichia coli* at different concentrations. The inhibition zone diameters obtained are presented in Table 4.

**Table 4:** Diameter of the inhibition zone (mm) of gentamicin at different concentrations against *S. aureus* and *E. coli*

*C*_GC_ a (mg mL^−1^)	GC1	GC2	GC3	GC4
	1	1.5	2	2.5
*D*_iz_ b (mm) of gentamicin against *S. aureus*	11	16.5	21.5	27
*D*_iz_ (mm) of gentamicin against *E. coli*	17	22	27.5	30.5

a *C*_GC_: gentamicin concentration.

b *D*_iz_: diameter of the inhibitory zone.

The results indicate that gentamicin exhibited antibacterial activity against both bacterial strains. However, its inhibitory effect was more pronounced against *E. coli* than against *S. aureus* at all tested concentrations. For instance, at a concentration of 2.5 mg mL^−1^, gentamicin produced inhibition zones of 30.5 mm for *E. coli* and 27 mm for *S. aureus*. This difference may be related to variations in bacterial cell structure and susceptibility to aminoglycoside antibiotics.

Although gentamicin demonstrated effective antibacterial activity, its performance remained lower than that observed for some onion seed oil extracts tested individually. This observation highlights the strong antimicrobial potential of the extracted oil and suggests that combining it with conventional antibiotics could further enhance antibacterial efficacy.

#### 3.5.2 Combined effect of gentamicin and onion seed oil

To evaluate the potential synergistic interaction between the antibiotic and the natural extract, mixtures of gentamicin at different concentrations and onion seed oil at its minimum inhibitory concentration (MIC) were tested against both bacterial strains. The results revealed a clear improvement in antibacterial activity when gentamicin was combined with onion seed oil. For *S. aureus*, the inhibition zone diameter increased from 27 mm for gentamicin alone (2.5 mg mL^−1^) to up to 30.5–32.5 mm when combined with the oil extract, depending on the extract used. This represents an enhancement of approximately 20% compared with the antibiotic alone (Table 5).

**Table 5:** Optimization of antibacterial efficacy against *S. aureus*: interactions between gentamicin at various concentrations and the MIC (0.25 mg mL^−1^) of onion seed oil extract

*C*_GC_ a (mg mL^−1^)	C1	C2	C3	C4
	1	1.5	2	2.5
*D*_iz_ b (mm) of gentamicin against *S. aureus*	11	16.5	21.5	27
*D*_iz_ (mm) of gentamicin–onion seeds oil (mm) (extract 1)	12.5	17.5	22.5	30.5
*D*_iz_ (mm) of gentamicin and onion seeds oil (extract 2)	—	12.5	22	32.5
*D*_iz_ (mm) of gentamicin and onion seeds oil (extract 3)	13.5	21	23	28.5

a *C*_GC_: gentamicin concentration.

b *D*_iz_: diameter of the inhibitory zone.

A similar trend was observed for *E. coli*. When gentamicin at 2.5 mg mL^−1^ was combined with onion seed oil at its MIC (0.25 mg mL^−1^), the inhibition zone diameter increased from 30.5 mm to values reaching approximately 37.5 mm. This corresponds to an increase of about 23% in antibacterial activity (Table 6).

**Table 6:** Optimization of antibacterial efficacy against *E. coli*: interactions between gentamicin at various concentrations and the MIC (0.25 mg mL^−1^) of onion seed oil extract

*C*_GC_ a (mg mL^−1^)	C1	C2	C3	C4
	1	1.5	2	2.5
*D*_iz_ b (mm) of gentamicin against *E. coli*	17	22	27.5	30.5
*D*_iz_ (mm) of gentamicin–onion seeds oil (mm) (extract 1)	17.5	22.5	29	37.5
*D*_iz_ (mm) of gentamicin and onion seeds oil (extract 2)	19	22.5	27	32.5
*D*_iz_ (mm) of gentamicin and onion seeds oil (extract 3)	17	27.5	28	33

a *C*_GC_: gentamicin concentration.

b *D*_iz_: diameter of the inhibitory zone.

These results demonstrate that onion seed oil can significantly enhance the antibacterial efficacy of gentamicin. The observed synergistic effect may be explained by several possible mechanisms. Plant-derived lipophilic compounds are known to interact with bacterial cell membranes, increasing membrane permeability and facilitating the penetration of antibiotics into bacterial cells. In addition, certain phytochemicals may inhibit bacterial resistance mechanisms such as efflux pumps or enzymatic degradation of antibiotics.

Such interactions between plant extracts and antibiotics have been previously reported in the literature. Natural bioactive compounds may act as resistance-modifying agents, restoring or enhancing the activity of conventional antimicrobial drugs.^37^ Overall, the results obtained in this study suggest that onion seed oil extracted by supercritical CO_2_ could serve as a promising natural adjuvant to improve the efficacy of gentamicin and potentially reduce the required antibiotic dosage.

### 3.6 FTIR analysis

The onion seed oil extracts obtained by supercritical CO_2_ extraction were characterized using Fourier transform infrared (FTIR) spectroscopy to identify their main functional groups and evaluate their potential interactions with gentamicin. The FTIR spectra of extracts 1, 2, and 3, gentamicin, and their corresponding mixtures are presented in Fig. 4, while the principal absorption bands and their vibrational assignments are summarized in Table 7.

**Fig. 4:**
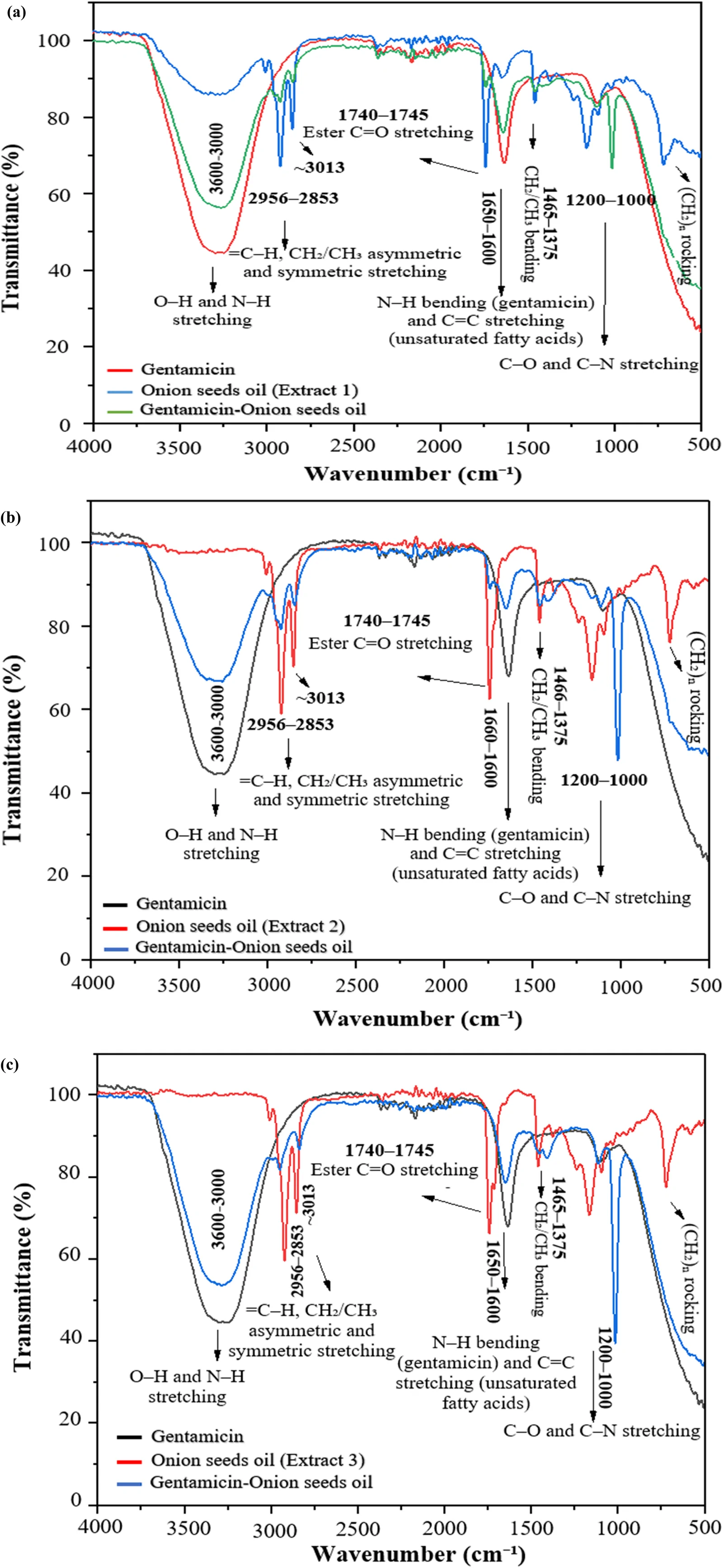
FTIR spectra of gentamicin, onion seed oil extract, and their mixture: (a) extract 1; (b) extract 2; (c) extract 3.

**Table 7:** Main FTIR absorption bands and their corresponding vibrational assignments for onion seed oil extracts, gentamicin, and their mixtures

FTIR band (cm^−1^)	Vibrational assignment	Main interpretation
3600–3000	O–H and N–H stretching	Hydroxyl groups from minor polar constituents of onion seed oil and hydroxyl/amino groups of gentamicin
∼3013	=C–H stretching	Olefinic (=C–H) stretching of *cis*-unsaturated fatty acids
2956–2853	CH_2_/CH_3_ asymmetric and symmetric stretching	Long aliphatic hydrocarbon chains of triacylglycerols
1740–1745	Ester C=O stretching	Ester carbonyl groups confirming the triacylglycerol-rich lipid matrix
1650–1600	N–H bending (gentamicin) and C=C stretching (unsaturated fatty acids)	N–H bending associated with gentamicin together with the contribution of C=C stretching from the unsaturated fatty acids present in onion seed oil
1465–1375	CH_2_/CH_3_ bending	Deformation vibrations of aliphatic lipid chains
1200–1000	C–O and C–N stretching	Ester C–O vibrations of lipids together with C–O/C–N vibrations of gentamicin
∼722	(CH_2_)_*n*_ rocking	Long-chain fatty acids
Extract–gentamicin mixtures	Retention of the characteristic bands with slight shifts and intensity variations; no new absorption bands	Indicates predominantly non-covalent interactions (possibly involving hydrogen bonding) without formation of new covalent bonds

Overall, the three extracts exhibited the characteristic FTIR features of a triacylglycerol-rich lipid matrix, including O–H, aliphatic C–H, ester C=O, C=C, and C–O vibrations.^38,39^ In contrast, gentamicin showed its characteristic O–H/N–H, C–O, and C–N absorption bands, consistent with its aminoglycoside structure.^40^

In the extract–gentamicin mixtures, the characteristic absorption bands of both components were retained, accompanied only by slight shifts in band position and minor variations in intensity, while no new diagnostic absorption bands were detected. These observations indicate that mixing did not induce major covalent chemical modifications and suggest that the interaction between the extracts and gentamicin is predominantly non-covalent.

### 3.7 TGA results

Fig. 5 shows the thermogravimetric (TG) curves of onion seeds before extraction (raw sample) and after supercritical CO_2_ extraction (residue). The masses are normalized (100% at the start of the test). Overall, both profiles show decomposition typical of a plant matrix (biomass), with limited initial mass loss, followed by a main devolatilization stage and then gradual carbonization at high temperatures.

**Fig. 5:**
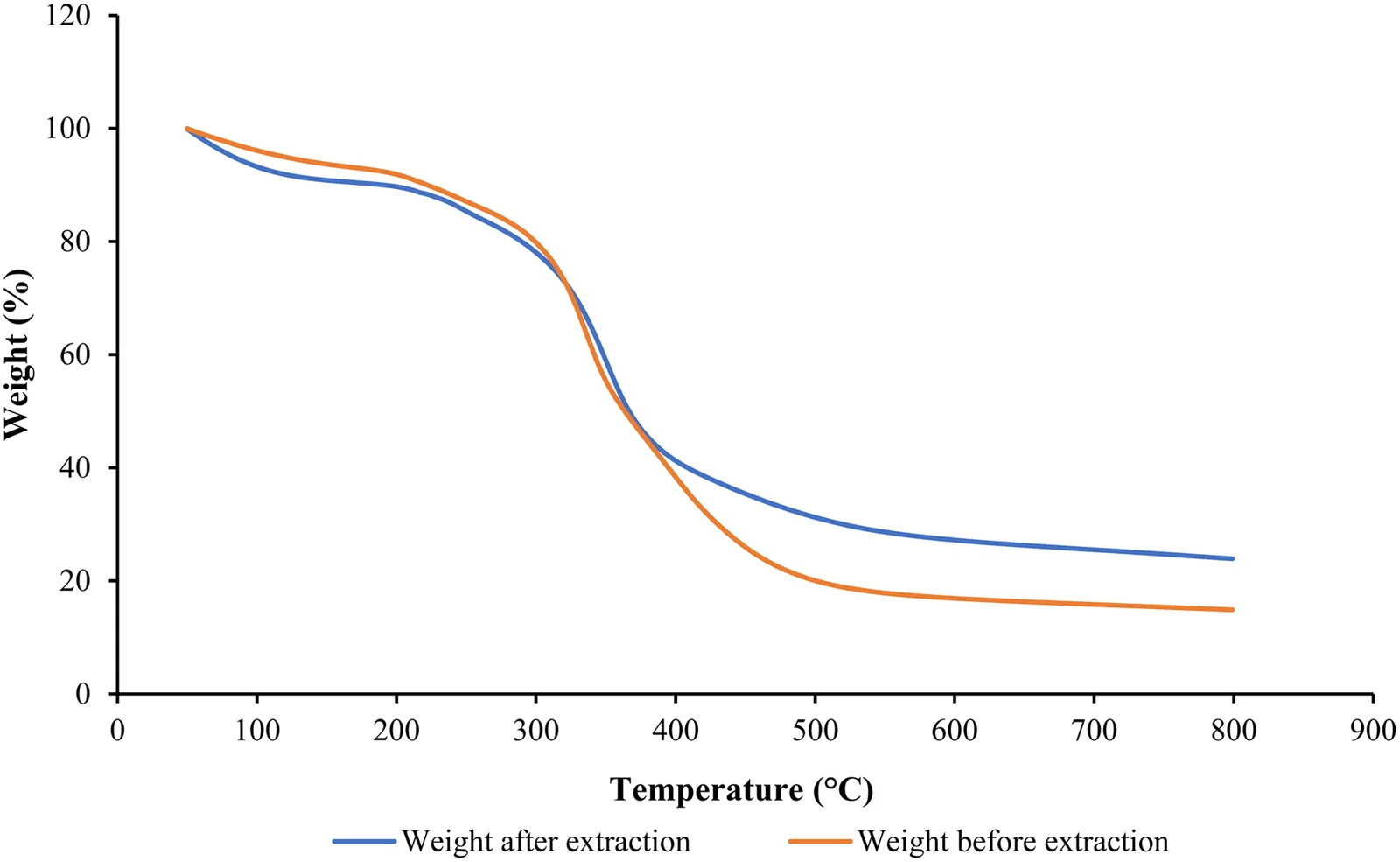
TGA profiles of onion seeds before and after supercritical CO_2_ extraction.

At low temperatures, below 150 °C, a slight mass loss is observed, which is mainly attributed to the desorption of free and weakly bound moisture, as well as the release of highly volatile compounds. The main mass loss occurs between 180 and 400 °C and corresponds to the thermal degradation of the principal organic constituents. According to the literature, hemicelluloses decompose at lower temperatures than cellulose, whereas lignin degrades more progressively over a wide temperature range. In seed materials, additional contributions from proteins and fatty acids, either free or esterified as triglycerides, may also enhance devolatilization within this temperature interval. Beyond 420 °C, the mass loss becomes slower and gradually approaches a final residue, which is associated with the formation of char (carbonaceous matrix) and the mineral fraction (ash). This slow evolution is consistent with the contribution of lignin and carbonization processes.^41–43^

The overall thermal degradation profile remains similar before and after supercritical CO_2_ extraction, with no evidence of additional degradation stages or marked changes in the main mass-loss regions. However, the extracted sample exhibits a higher residual mass at 800 °C (23%) than the raw sample (18%). This increase is attributed to the removal of extractable lipids during the supercritical CO_2_ extraction process. Since lipids mainly decompose into volatile products during heating and contribute only marginally to char formation, their removal increases the relative proportion of structural polymers and mineral constituents in the remaining solid matrix, resulting in a higher final residual mass.^44^ Therefore, the TGA results primarily reflect compositional changes induced by lipid extraction rather than a fundamental modification of the thermal degradation behaviour of the seed matrix.

### 3.8 SEM analysis of onion seeds before and after supercritical CO_2_ extraction

#### 3.8.1 Morphology prior to extraction

Onion seeds appear as relatively compact lamellar fragments, with generally continuous and cohesive surfaces. The surfaces of the particles are moderately rough, with no pronounced cracks or open cavities. This observation indicates that the plant matrix remains closed and structurally intact, reflecting limited mechanical alteration of the raw material (Fig. 6(a)).

**Fig. 6:**
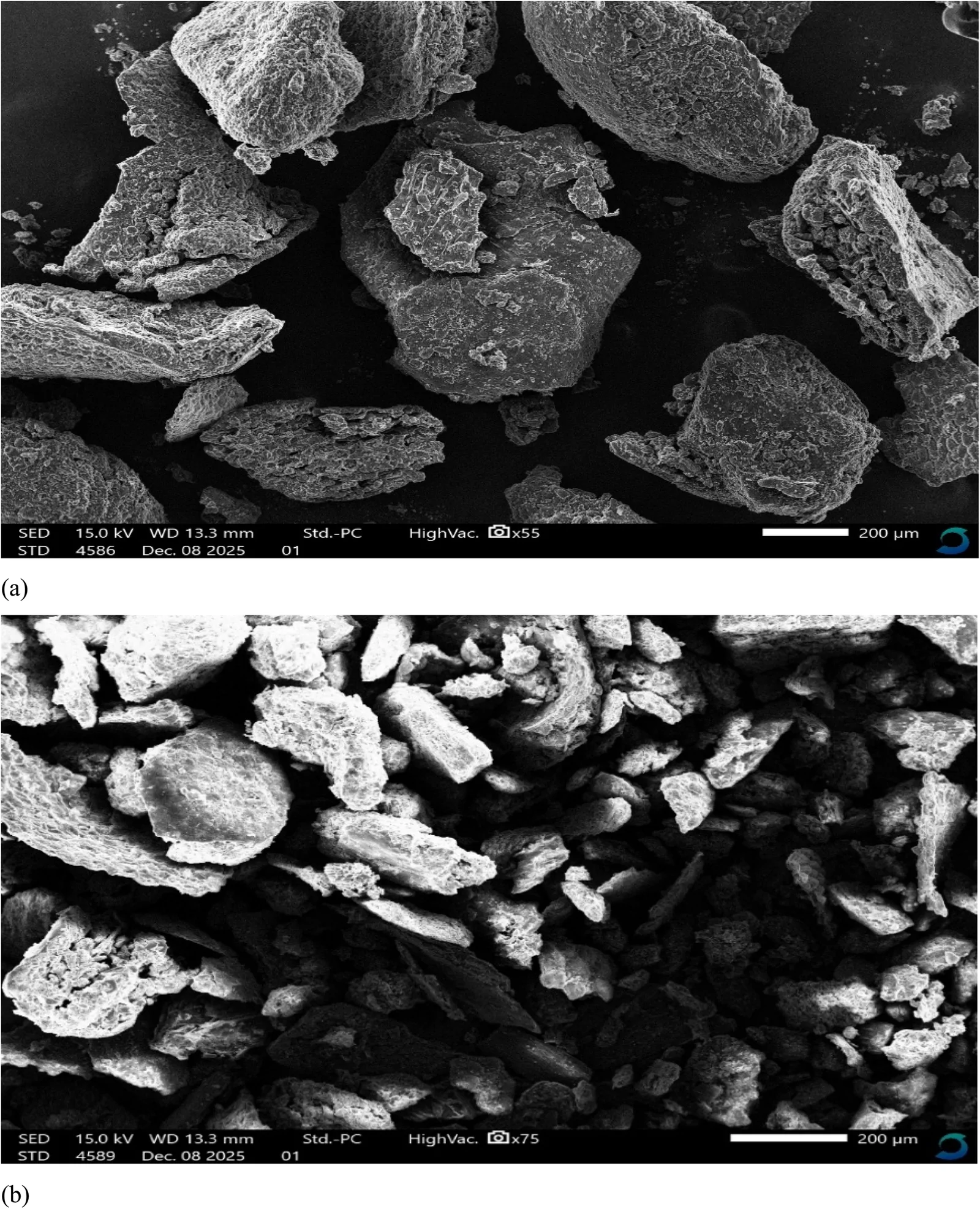
SEM micrographs of onion seeds: (a) seeds before supercritical CO_2_ extraction; (b) solid residue after supercritical CO_2_ extraction.

#### 3.8.2 Morphology after supercritical CO_2_ extraction

Once supercritical CO_2_ extraction is complete, significant morphological changes can be observed. The solid residue is more fragmented, with finer debris and a visibly less cohesive structure (Fig. 6(b)). The surfaces become rougher and more fragile, with the appearance of microcracks, cavities, and eroded areas, indicating a significant loss of compactness. These changes reflect a partial disruption of the internal structure of the plant matrix.

#### 3.8.3 Effects of supercritical CO_2_ penetration and extraction of soluble compounds

Due to its high diffusivity and low viscosity, CO_2_ penetrates the plant matrix effectively and promotes the extraction of soluble compounds.

The removal of these components leads to the formation of structural voids that weaken the solid matrix and promote fragmentation of the material.

## 4. Conclusion

This study demonstrated the potential of onion seed oil extracted by supercritical CO_2_ as a natural agent capable of enhancing the antibacterial activity of the antibiotic gentamicin. The results showed that the oil extracts contained bioactive compounds such as phenolics, flavonoids, and tannins, which may contribute, together with other constituents, to the observed biological activity.

The antibacterial assays revealed significant inhibitory effects of the onion seed oil against *Staphylococcus aureus* and *Escherichia coli*.

Furthermore, the combination of onion seed oil with gentamicin produced a clear synergistic effect, leading to an increase in the inhibition zone diameters of up to approximately 20% for *S. aureus* and 23% for *E. coli*. This enhancement may be related to interactions between lipophilic compounds present in the oil and bacterial cell membranes, which could facilitate antibiotic penetration into bacterial cells.

The physicochemical analyses, including FTIR, TGA, and SEM, confirmed the lipidic nature of the extracts and provided insight into the structural and thermal properties of the onion seed matrix before and after extraction. Overall, the present study demonstrates that supercritical CO_2_ extraction can provide onion seed oil with substantial antibacterial activity and can enhance the inhibitory effect of gentamicin against both *S. aureus* and *E. coli*. Among the investigated extraction conditions, an oil yield of 24.35% and a total phenolic content of 36.15 mg GAE per g were obtained. Compared with previously reported conventional extraction approaches for onion seeds, these values are competitive in terms of oil recovery and phenolic compounds, with reported oil yields of 13.50% for Soxhlet extraction and 21.10% for cold pressing, and total phenolic contents of 1.84–6.87 and 3.35 mg GAE per g, respectively.^18,45^

Although these values originate from different studies and experimental conditions and therefore do not constitute a direct experimental comparison, they provide useful evidence for contextualizing the performance of supercritical CO_2_ extraction. In addition to its extraction performance, supercritical CO_2_ offers advantages such as the absence of conventional organic solvents and the possibility of controlling extraction selectivity through pressure and temperature, while avoiding prolonged exposure of the extracted compounds to elevated temperatures.^46^ Nevertheless, the technique requires high-pressure equipment and entails higher initial investment, and the extraction of more polar compounds may require the use of co-solvents. Therefore, supercritical CO_2_ should be considered a promising alternative rather than a universally superior replacement for conventional extraction methods. In the present study, its potential lies in combining efficient oil recovery and phenolic compound recovery with the production of an extract exhibiting antibacterial activity and an enhanced effect when used in combination with gentamicin. A major limitation of the present study is the absence of compound-resolved chromatographic and mass-spectrometric characterization, particularly LC-MS/MS analysis. The colorimetric assays used here provide global estimates of phenolic, flavonoid, and hydrolysable tannin fractions but do not permit identification of the individual compounds responsible for the observed antibacterial activity. Therefore, the biological effects cannot be attributed to specific constituents without further molecular characterization. Further studies should therefore focus on detailed LC-MS/MS profiling to identify the specific active compounds involved in the antibacterial response and in the enhanced effect observed in combination with gentamicin.

## Conflicts of interest

There are no conflicts to declare.

## Data Availability

All data supporting the finding of this study are contained within the manuscript and its supplementary information (SI) file. Additional data are available from the corresponding authors upon reasonable request. Supplementary information is available. See DOI: https://doi.org/10.1039/d6ra04660f.
